# Editorial: Nutritional therapy along the continuum of care

**DOI:** 10.3389/fnut.2024.1398632

**Published:** 2024-07-02

**Authors:** Barbara Troesch, Matthias Pirlich

**Affiliations:** ^1^Barbara Troesch Scientific Writing, Zurich, Switzerland; ^2^Imperial Oak Outpatient Clinic, Berlin, Germany

**Keywords:** clinical nutrition, nutrition therapy, Human Right to Nutritional Care, GLIM criteria, nutritional screening, nutritional assessment

At the 2022 ESPEN conference, the International Declaration on the Human Right to Nutritional Care was presented, stating the ethical obligation to ensure optimal nutritional care (1). Increasing evidence supports the beneficial impact that nutritional therapy has on outcomes such as mortality and hospital readmission (2). Given this clear call to action, the objective of this Research Topic on “*Nutritional therapy along the continuum of care*” is to bring together relevant research that contribute to making it a reality. Nutritional therapy can include a range of interventions such as dietary counseling or oral, enteral, or parenteral clinical nutrition as well as a combination of these. In addition, nutritional interventions need to be part of a holistic treatment plan in combination in-depth anamneses, medical interventions, physical activity as well as other forms of therapy.

The International Declaration on the Human Right to Nutritional Care states that “*right to food is often overlooked in the clinical setting, resulting in an unacceptable number of children and adults suffering from disease-related malnutrition in hospitals and in the community, leading to an unacceptable disregard of the right to health*” (1). To address this, it is important to develop a common understanding of the definition and classification of malnutrition. A further narrative review summarized the evolution of the diagnosis of malnutrition during the last two decades (Hegazi et al.). A special emphasis is Global Leadership Initiative on Malnutrition (GLIM) that aimed at creating a consensus on criteria for the diagnosis of malnutrition (3). The authors in the current Research Topic propose an approach that integrates the GLIM criteria into the WHO frameworks and considers different forms of malnutrition in both adult and pediatric populations (Hegazi et al.).

One of the two key pillars of the Human Right to Nutritional Care is the Human Right to Food (1), relevant before a person even enters the healthcare system. In our aging populations, patients often present with a range of chronic conditions that, in combination with poor lifestyle choices and other factors such polypharmacy, affect their nutritional status (4). Often hidden behind adipose tissue, their muscle mass is decreased (5), and intakes of essential nutrients are low (6), while inflammatory levels are chronically increased.

In addition, socio-economic factors affect their ability to maintain an adequate nutritional status as seen in two cohort studies presented here: The analysis of a Swiss sub cohort of the EFFORT trial with 433 elderly patients shows that even in a country with a high level of affluence and a relatively efficient social security system, 6.9% were food insecure (Rigling et al.). Age, dependence on welfare, and loneliness were significant factors associated with food insecurity, which in turn was linked to a significantly lower quality of life. In the other cohort study presented here, lower levels of food security (defined through educational level and income) were significantly associated with a higher risk of malnutrition (Ouaijan et al.). Among the >340 patients from five hospitals in Lebanon, the malnutrition prevalence was 35.6% based on the Global Leadership Initiative for Malnutrition (GLIM) criteria [(3), Ouaijan et al.]. Addressing nutritional care in the community is the topic of a recent ESPEN publication (7) and goes beyond the scope of this editorial.

Consequently, patients often enter the hospital malnourished, they continue to lose muscle mass during the stay, and they fail to recover it after discharge. Decrease in food intake due to factors such as lack of exercise, stress caused by the hospital stay and metabolic changes because of their medical condition, surgery, or drugs, further aggravates the macro- and micronutrient deficiencies (8). All of this affects their clinical prognosis and the increase in frailty puts them at risk of further health problems, leading to a vicious cycle of malnutrition, ill health, and frailty.

The significant prognostic impact of malnutrition regarding increased in-hospital mortality and longer ICU stay was demonstrated in a large cohort of elderly patients in a cardiac intensive care unit in China (Li Y. et al.). Another study from China showed the prognostic importance of body composition analysis in a large cohort of cancer patients (Ji et al.). About a quarter of all examined cancer patients had reduced muscle mass, which was significantly associated with lower survival. In a cohort study of over 160,000 intensive care patients in China, the obesity paradox concerning cardiovascular mortality was confirmed (Li S. et al.). The lowest mortality rate was observed in mild obesity, but in severely obese and underweight individuals, the mortality was pronounced.

The recent Corona virus 2019 (COVID-19) pandemic taught us that the relationship between different risk and/or prognostic factors can be complex, which also applies to malnutrition: A narrative review in this Research Topic addresses the two major risk factors for an unfavorable course of COVID-19: diabetes mellitus 2, and malnutrition (Mechanick et al.). They postulate a syndromic triad and suggest ways for early preventive care through nutritional and lifestyle interventions.

Following screening and assessment of malnutrition, an individual nutritional care needs to be established to ensure adequate intake of energy and protein as well as other essential nutrients. Oral Nutritional Supplements (ONS) have been shown to be a useful means to improve nutritional status, particularly if they were energy dense (9). This finding is supported by a randomized controlled trial published in this Research Topic: the intervention in an outpatient setting demonstrated that an energy dense ONS (2.4 kcal/ml) was well-tolerated and was non-inferior to high-energy ONS (2.0 kcal/ml) in malnourished patients, allowing for more calories in a lower volume (Leon-Sanz et al.).

Depending on the underlying disease or population group, the nutritional therapy needs to be adjusted in factors other than energy density: A retrospective study on 63 patients found evidence that the application of a medium-chain fatty acid diet might be effective in treating post-operative chylous leakage (Wang et al.). The possible benefits of a starched thickened formula with reduced lactose content and pre- and probiotic ingredient were demonstrated in young infants with regurgitations and colic (Chouraqui et al.).

According to the declaration, clinical nutrition education is another essential factor for the implementation of optimal nutritional care (1). Another study investigated the structures and current practices of nutritional support in Saudi Arabian hospitals (Ajabnoor et al.). Of the 114 participating physicians, pharmacists, and dietitians, only 44.7% reported working with a formal nutritional support team. The unsurprising finding that confidence in using nutritional interventions such as enteral nutrition was associated with nutritional qualification, highlights the key role of dietitians in implementing optimal nutritional care (Ajabnoor et al.). However, it also showed that nutritional care improved if other key stakeholders, such as physicians, were involved (Ajabnoor et al.).

The respect of patient dignity is an important aspect when decisions on optimal nutrition are taken, particularly in a palliative setting at the end of life. It was recommended that nutritional therapy should become less invasive as life expectancy decreases and focus increasingly on relieving eating-related distress and thirst (10). Particularly the use of parenteral nutrition is seen as controversial in these situations, but prognosis of life expectancy is difficult. In this special edition, a Viennese working group examined whether the clinical benefit of parenteral nutrition in a palliative situation can be predicted by an algorithm based on laboratory parameters, which needs further validation (Kum et al.).

In summary, this special edition provides new insights into the effect of malnutrition on patient's clinical prognosis and quality of life as well as potential solutions to improve nutritional interventions along the continuum of care. Moreover, it serves as a call for action to close gaps in our understanding of the problem and for implementation of further initiatives to optimize nutritional therapies.

## Author contributions

BT: Conceptualization, Writing – original draft, Writing – review & editing. MP: Conceptualization, Writing – original draft, Writing – review & editing.
